# Clinical Efficacy of Early and Late Vedolizumab or Infliximab Interventions in Moderate Ulcerative Colitis: A Multicenter, Retrospective Cohort Study

**DOI:** 10.14740/gr2092

**Published:** 2026-01-04

**Authors:** Rui Ping Meng, Jian Zhang, Hao Qi Wei, Xi Ping Liao, En Liu, Hui Lin, Bao Bao Huang, Lin Lyu, Yan Ling Wei, Jian Yun Zhou, Xia Xie

**Affiliations:** a Department of Gastroenterology, the Second Affiliated Hospital of the Army Medical University, Chongqing 400037, China; b Department of Cardiology, Fuwai Yunnan Cardiovascular Hospital, Yunnan 650032, China; c Center of Teaching and Training, DaPing Hospital of Army Medical University, Chongqing 400042, China; d Department of Gastroenterology, the Second Affiliated Hospital of Chongqing Medical University, Chongqing 400060, China; e Department of Gastroenterology, DaPing Hospital of Army Medical University, Chongqing 400042, China; f Clinical Medical Research Center, the Second Affiliated Hospital of the Army Medical University, Chongqing 400037, China; g Department of Gastroenterology, the PLA 77th Group Army Hospital, Sichuan 614000, China

**Keywords:** Early biologic intervention, Late biologic intervention, Moderate ulcerative colitis, Efficacy

## Abstract

**Background:**

Late biologic intervention has been partly displaced during the last decade by early biologic therapy. However, there is a scarcity of direct evidence to inform clinical decision-making with greater confidence. The study aimed to compare the efficacy and safety of the early biologic approach with those of the late biologic approach in patients with moderate ulcerative colitis (UC).

**Methods:**

Moderate UC patients treated with biologics between January 2021 and February 2024 from three Chinese centers were retrospectively included. The outcomes included steroid-free clinical remission rates, clinical remission rates, and mucosal healing rates at week 14 and week 52.

**Results:**

A total of 124 moderate UC cases were included. No marked differences in the steroid-free clinical remission rates and clinical remission rates were observed between the two groups at week 14 or at week 52 (P > 0.050). The early biologic therapy group exhibited a numerically higher mucosal healing rate at week 14 (23.3% vs. 12.5%, P = 0.115) and week 52 (15/27 (55.6%) vs. 13/37 (35.1%), P = 0.104) compared to the late biologic therapy group, yet there was no significant difference between two groups.

**Conclusion:**

No significant differences in steroid-free clinical remission, clinical remission, and mucosal healing were observed between early and late biologic intervention group.

## Introduction

Ulcerative colitis (UC) is a recurrent inflammatory disease characterized by progressive mucosal inflammation that initiates in the rectum and potentially extends to proximal colon segments [1]. The prominent clinical symptoms are persistent or recurrent bloody diarrhea, abdominal discomfort, urgency, and tenesmus, sometimes accompanied by peripheral arthritis, oral ulcers, erythema nodosum, iritis, primary sclerosing cholangitis, and other extraintestinal manifestations [2, 3].

Traditional UC therapeutic regimens include 5-aminosalicylic acid (5-ASA), corticosteroids, and immunomodulators. The advent of biologic agents that can facilitate specific immune modulation has broadened the therapeutic horizon for patients with varied drug history profiles. Nevertheless, biologics are often reserved for cases in which UC patients have high risk factors or exhibit traditional therapy ineffectiveness or intolerance [4, 5]. In such a situation, late biologic intervention may amplify complications, hospitalizations, and colectomies and deteriorate quality of life. Moreover, this approach potentially leads to excess corticosteroid consumption, thereby increasing the risk of side effects, such as osteoporosis, obesity, and elevated blood sugar, and approximately 50% of patients receiving steroids experience side effects [6, 7]. Moreover, increasing evidence shows that UC should be considered a disease with a progressive nature associated with adverse consequences such as Crohn’s disease (CD), so it is reasonable to treat UC early to alter the disease course and prevent complications [8-12]. In the meantime, evidence suggests that there is an expanding tendency to prescribe biologics for patients with UC, as manifested by the early intervention with biologics in patients’ disease course [13]. However, although the strategy of early intervention has accumulating evidence in CD, there is less evidence supporting its impact on UC [10, 13], especially in those with moderate UC.

Therefore, we urgently need a large amount of evidence to verify whether early intervention with biologics can achieve a better prognosis than late. The current multicenter, retrospective cohort study was performed to compare the efficacy and safety of early intervention with biologics with those of late biologic initiation in moderate UC patients.

## Materials and Methods

### Study design and patients

We carried out a multicenter, retrospective observational study at three Chinese hospitals in Southwest China between January 2021 and February 2024. The inclusion criteria were as follows: 1) age ≥ 18 years; 2) diagnosis of moderate UC (total Mayo score ranging from 6 to 10 at baseline); 3) treatment with vedolizumab (VDZ) or infliximab (IFX) for at least 14 weeks; and 4) patients who have not received any biological agents. The exclusion criteria were as follows: 1) had a history of colectomy and 2) were pregnant.

The pharmacotherapy regimen was as follows: three 300 mg infusions of VDZ were prescribed at weeks 0, 2, and 6, with subsequent infusions of the same dosage every 8 weeks. IFX was started by intravenous infusion of 5 to 10 mg/kg at weeks 0, 2, and 6, followed by the analogous dose every 8 weeks.

### Data collection

**Figure 1:**
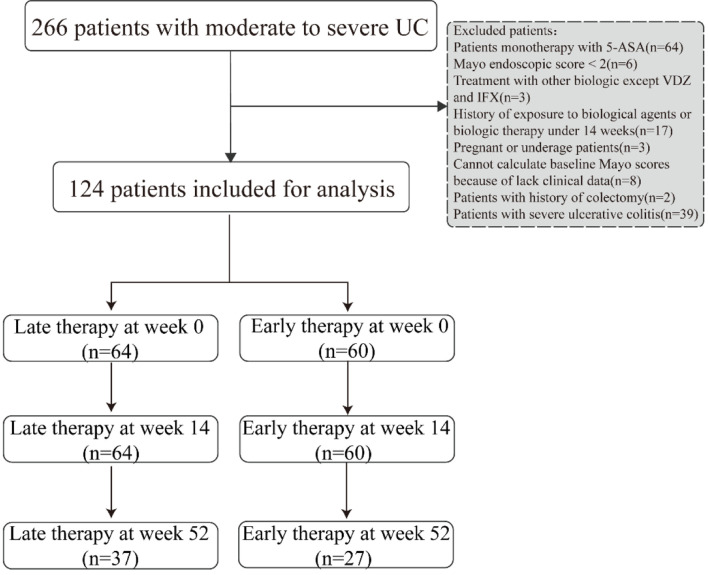
Study flowchart of patients included in analysis.

Patients were divided into late intervention group and early intervention therapy group according to the therapeutic strategy (Fig. 1). The following data at inclusion were collected through a standardized form: 1) basic information: age, sex, body mass index (BMI), smoking history, disease duration, and disease extent according to the Montreal classification [14]; 2) medical history: 5-ASA, hormone or immunosuppressive; 3) clinical characteristics: clinical symptoms, Mayo endoscopic scores, Mayo scores, and disease severity according to Mayo scores [15]; 4) inflammatory and nutritional indicators: hypersensitive C-reactive protein (hs-CRP), erythrocyte sedimentation rate (ESR), platelet (PLT), hemoglobin (Hb), albumin (ALB) levels, etc.

### Follow-up

The follow-up period began with the initial use of biologics until death, colectomy, discontinuation of treatment, or the end of the follow-up period in May 2024. During the follow-up period, the following information was collected at each treatment administration: dose and frequency of biologics, concomitant therapy drugs, clinical symptoms, biological variables (hs-CRP, ESR, PLT, Hb, and ALB levels), and available endoscopic data. Treatment optimization, adverse events (infusion reactions, concomitant infections, immune system diseases, tumors, colectomy, death, etc.), and the reasons for treatment discontinuation were also recorded during the follow-up period.

### Objectives and definitions

The outcome evaluation indices included: 1) the steroid-free clinical remission rates, clinical remission rates, and mucosal healing rates at 14 and 52 weeks; 2) the Mayo score, inflammatory indices, and nutritional indices at 14 weeks; 3) the time to achieve clinical remission; and 4) adverse events. Steroid-free clinical remission was defined by a partial Mayo score (stool frequency plus blood in stool) < 2 and no use of any form of hormone for at least 4 weeks [16, 17]. Clinical remission was defined by a normal stool frequency and a bleeding subscore of 0 [18]. Mucosal healing was defined by a Mayo endoscopic score of 0 [18].

Early biologic treatment was defined as disease duration ≤ 36 months (determined by time since diagnosis, not symptom onset) and no prior or current treatment with disease-modifying drugs (e.g., steroids, immunomodulators or biologics). Conversely, late biologic therapy was defined by disease duration > 36 months or exposure to steroids or immunomodulators before first biological treatment [19].

Treatment optimization included increasing the infusion frequency or dose of biologics [20].

### Statistical analysis

Data analyses were performed using SPSS 25.0. Since there was only a small amount of missing data for BMI, ESR, PLT, and ALB (< 5%), we used the method of multiple interpolation to address the missing values. The Shapiro-Wilk test was used to test whether the continuous variables conformed to a normal distribution. Variables that conformed to a normal distribution are reported herein as the mean ± standard deviation (SD) and were analyzed using Student’s *t*-test; otherwise, variables were analyzed using the Mann-Whitney U test and are reported as medians (interquartile ranges). Categorical data are presented as percentages and were analyzed using the Chi-square test and Fisher’s exact test. All analyses used two-sided statistical tests. P < 0.05 was considered the threshold for significance.

Kaplan-Meier survival curves were generated to demonstrate the clinical remission rates for each treatment strategy over time, and significant differences were compared with the log-rank test.

### Ethical statements

This study was conducted with respect to the requirements set out in the applicable standard operation procedures of the Medical Ethics Committee of the Second Affiliated Hospital of Chinese People’s Liberation Army (PLA) Medical University (2023-Research No. 080-01).

## Results

### Baseline characteristics

Our study cohort included 124 patients with moderate UC. The mean age of the enrolled patients was 43.8 years, 63.7% were men, and the mean disease duration was 56.3 months. With respect to disease extent, 47 (37.9%) had left-sided disease, and 77 (62.1%) had pancolitis. The subjects were divided into a late biologic treatment group (n = 64) and an early biologic therapy group (n = 60) according to the therapeutic strategy that they received. There was no significant difference in baseline parameters between the late initiation group and early intervention group (Table 1).

**Table 1:** Population Characteristics at Baseline Stratified by Treatment Strategy

Baseline matching variables	Late (n = 64)	Early (n = 60)	P value
Gender, male, n(%)	40 (62.5%)	39 (65.0%)	0.772
Age, years, median (IQR)	47.0 (30.3 - 58.0)	40.5 (32.0 - 56.8)	0.687
BMI, kg/m^2^, mean ± SD	20.5 ± 3.2	21.1 ± 3.2	0.310
History of smoking, n (%)	16 (25.0%)	13 (21.7%)	0.661
Extraintestinal manifestation, n (%)	1 (1.6%)	4 (6.7%)	0.324
Disease extent, n (%)			0.310
E2	27 (42.2%)	20 (33.3%)	
E3	37 (57.8%)	40 (66.7%)	
Mayo score, median (IQR)	8.0 (6.3 - 9.0)	8.0 (7.0 - 9.0)	0.224
ESR, mm/h, median (IQR)	20.5 (6.1 - 37.9)	26.5 (11.5 - 38.8)	0.261
hs-CRP, mg/L, median (IQR)	4.6 (0.7 - 11.4)	5.5 (1.9 - 37.7)	0.089
PLT, × 10^9^/L, median (IQR)	261.0 (209.3 - 338.3)	301.5 (214.3 - 406.0)	0.224
Hb, g/L, median (IQR)	125.0 (110.5 - 139.5)	127.0 (107.0 - 138.0)	0.818
ALB, g/L, median (IQR)	40.3 (36.3 - 44.8)	38.5 (32.2 - 43.5)	0.103
Biologics in treatment, n (%)			0.953
VDZ	27 (42.2%)	25 (41.7%)	
IFX	37 (57.8%)	35 (58.3%)	
Concomitant medications, n (%)			
Steroid	7 (10.9%)	5 (8.3%)	0.624
Immunosuppressant	12 (18.8%)	6 (10.0%)	0.167

ALB: albumin; BMI: body mass index; ESR: erythrocyte sedimentation rate; Hb: hemoglobin; hs-CRP: hypersensitive C-reactive protein; IFX: infliximab; IQR: interquartile range; PLT: blood platelet; SD: standard deviation; VDZ: vedolizumab.

### Effectiveness evaluation at week 14

**Figure 2:**
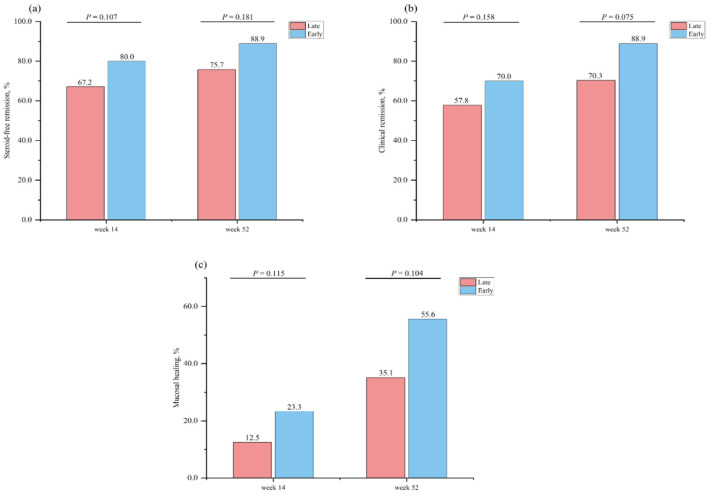
Proportion of patients in steroid-free remission at week 14 and week 52 stratified by therapeutic strategy (a). Proportion of patients in clinical remission at week 14 and week 52 stratified by therapeutic strategy (b). Proportion of patients in mucosal healing at week 14 and week 52 stratified by therapeutic strategy (c).

Steroid-free clinical remission at week 14 was achieved in 43/64 (67.2%) in the early biologic intervention group versus 48/60 (80.0%) in the late biologic initiation group (P = 0.107). The corresponding rates of clinical remission were 37/64 (57.8%) and 42/60 (70.0%), respectively (P = 0.158). In the early biologic treatment group, 14/60 (23.3%) patients had mucosal healing versus 8/64 (12.5%) in the late biologic therapy group (P = 0.115) (Fig. 2).

With respect to treatment optimization before week 14, the proportion of patients who experienced treatment optimization was similar between the late therapy group and the early therapy group (4.7% (3/64) vs. 5.0% (3/60), P > 0.999). Among the three patients who received late biologic treatment, two patients received an additional biologic infusion at week 10, and one patient received an additional biologic infusion at week 1. Among the three patients who received early biologic treatment, two patients increased their infusion dosage from week 1 and week 10 and one patient received an increased infusion dosage beginning at week 6.

Additionally, we found that the Mayo score, nutritional metrics, and inflammatory indices did not significantly differ (P > 0.05) (Table 2).

**Table 2:** Mayo score, inflammatory index and nutritive index at week 14.

	Late	Early	P value
Mayo score, median (IQR)	3.0 (2.0 - 6.0)	2.0 (1.0 - 5.8)	0.266
ESR, mm/h, median (IQR)	9.0 (5.0 - 27.0)	7.0 (5.0 - 15.0)	0.276
hs-CRP, mg/L, median (IQR)	1.3 (0.5 - 6.3)	0.9 (0.5 - 3.0)	0.110
PLT, × 10^9^/L, median (IQR)	217.0 (178.5 - 313.0)	223.5 (183.0 - 283.0)	0.993
Hb, g/L, median (IQR)	132.0 (115.5 - 145.5)	130.5 (116.0 - 149.0)	0.910
ALB, g/L, median (IQR)	43.1 (39.6 - 46.6)	44.2 (40.9 - 47.4)	0.360

ALB: albumin; ESR: erythrocyte sedimentation rate; Hb: hemoglobin; hs-CRP: hypersensitive C-reactive protein; IQR: interquartile range; PLT: blood platelet.

### Effectiveness evaluation at week 52

In total, 51.6% of patients (64/124) completed 52 weeks of follow-up in the cohort (Table 3). The mean follow-up duration of the enrolled patients was 37.9 weeks. The proportion of patients treated with the early biologic strategy who achieved clinical remission at week 52 was greater than that of the late biologic therapy patients, but this difference was not significant (26/37 (70.3%) vs. 24/27 (88.9%), P = 0.075). A similar picture was observed for steroid-free clinical remission and mucosal healing, which was numerically greater for early biologic therapy than for late biologic therapy but also did not significantly differ (28/37 (75.7%) vs. 24/27 (88.9%), P = 0.181) (13/37 (35.1%) vs. 15/27 (55.6%), P = 0.104) (Fig. 2).

**Table 3:** Assessment at Follow-Up Endpoint

	Late	Early
Treatment discontinuance		
Symptom improvement	1	3
Allergic reaction	1	2
Infection	1	1
Secondary nonresponse	1	-
Change medication		
Treatment failure	3	7
Abnormal liver function	-	1
Loss to follow-up	16	12
Colectomy	1	1
At the end of follow-up but less than 52 weeks	3	6

We found that the ESR level of the early biologic therapy group after 52 weeks of treatment was significantly lower than that of the late biologic therapy group (13.0 vs. 6.0 mm/h, P = 0.021). However, the Mayo score, nutritional metrics, and other inflammatory indices did not significantly differ (P > 0.05) (Table 4).

**Table 4:** Mayo Score, Inflammatory Index, and Nutritive Index at Week 52

	Late	Early	P value
Mayo score, median (IQR)	2.0 (1.0 - 5.0)	2.0 (1.0 - 4.0)	0.737
ESR, mm/h, median (IQR)	13.0 (8.0 - 22.0)	6.0 (4.0 - 11.0)	0.021
hs-CRP, mg/L, median (IQR)	1.1 (0.5 - 5.6)	1.0 (0.5 - 4.6)	0.738
PLT, × 10^9^/L, median (IQR)	215.5 (174.8 - 319.0)	245.5 (191.8 - 283.5)	0.802
Hb, g/L, median (IQR)	138.5 (119.3 - 149.8)	147.0 (134.5 - 155.5)	0.118
ALB, g/L, mean ± SD	43.3 ± 4.7	43.9 ± 3.2	0.672

ALB: albumin; ESR: erythrocyte sedimentation rate; hs-CRP: hypersensitive C-reactive protein; Hb: hemoglobin; IQR: interquartile range; PLT: blood platelet; SD: standard deviation.

These frequencies did not differ significantly from the frequency seen in patients who experienced treatment optimization between the late biological management group and the early biologic treatment group (5.4% (2/37) vs. 7.4% (2/27), P > 0.999). Among the two patients who received early biologic treatment, one patient increased their infusion dosage from week 18 and week 24 and one patient received an increased infusion dosage beginning at week 38. Two patients increased their infusion dosage from week 22 in the late biologic therapy group.

### Kaplan-Meier curve of clinical remission

**Figure 3:**
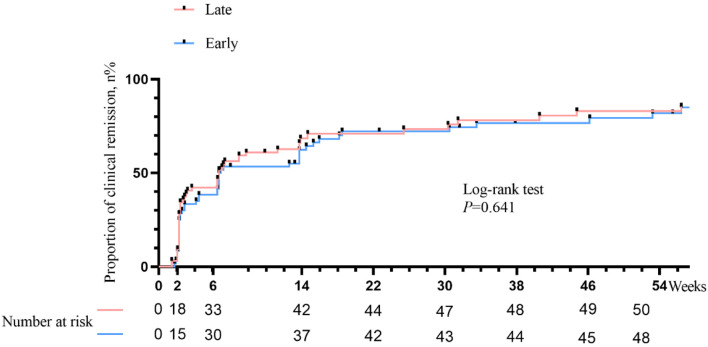
Kaplan-Meier curves of time to clinical remission stratified by therapeutic strategy.

The time to achieve clinical remission in late biologic therapy versus early biologic management during the follow-up period is shown in Figure 3. There was not a significant difference in the time to reach clinical remission in favor of early biologic treatment. The median time for achieving clinical remission with both therapies was 42 days (P = 0.641).

### Safety data

In total, there were 4.7% (3/64) documented adverse events in the late biologic therapy group (one *Clostridium difficile* infection and two allergic reaction) and 8.3% (5/60) in the early intervention group (three allergic reactions, one viral pneumonia, and one abnormal liver function); these differences were not significant (P = 0.645). Moreover, during the follow-up period, one patient in the late biologic management group underwent colectomy intervention, but none for the early biologic therapy group (1.6% (1/64) vs. 0.0% (0/60), P > 0.999).

## Discussion

The guideline has recommended that for patients with moderate to severe UC, early intervention with biologics is more preferable [21]. Therefore, we would like to conduct this study to explore whether this advantage is more pronounced in patients with moderate UC. To date, there is no specific guidance on how best to define early biologic intervention in UC or early UC [10, 12].

In our study, early intervention with biologics was defined as disease duration shorter than 3 years and the upfront use of biologics in patients with moderate UC, and patients in the late biologic treatment group had a disease duration greater than 3 years or had been exposed to steroids or immunomodulators before initiation of biological therapy. There were no statistically significant differences in the rates of steroid-free clinical remission, clinical remission, and mucosal healing at both weeks 14 and 52 between two groups.

Similarly, more studies have defined early intervention with biologics based on disease duration, and these studies indicate that early intervention has a negligible impact on UC [12, 19, 22-26]. For example, previous research by Faleck et al demonstrated no significant divergence in remission rates at 6 months between UC patients treated with VDZ who exhibited a disease duration of ≤ 2 years versus those with a longer disease duration [24]. Consistent with the conclusions of previous studies, after sensitivity analysis, even if the threshold is set at 2 years, there is still no statistical difference (Supplementary Material 1, gr.elmerpub.com). Similarly, another retrospective study revealed no notable differences in clinical and colectomy outcomes between early and late initiators of anti-tumor necrosis factor (anti-TNF) therapy within the first 3 years after diagnosis [25]. Furthermore, subanalysis of the ACT 1 and ACT 2 trials could not discern any difference in response rates to IFX in patients with disease durations less than or more than 3 years [23]. Unlike CD, the cutoff value employed has no influence on the duration of UC disease or on the efficacy of biological agents, regardless of the threshold used. Meanwhile, previous studies have also found that UC patients receiving anti-TNF or VDZ therapy had similar clinical outcomes to those of late initiators [24, 26, 27].

Mucosal healing is a crucial therapeutic goal in UC [9, 28, 29], and is associated with long-term clinical remission, lower incidences of clinical relapse, the need for hospitalization and surgery, and reduced rates of dysplasia and colorectal cancer. In achieving mucosal healing, biologics were rated significantly better than all other classes (P < 0.05) [5]. Thus, theoretically, earlier use of biologics could lead to mucosal healing and have a long-term benefit. According to our study, we observed that although this difference was not significant, there were numerically higher mucosal healing rates at week 14 and week 52 for early intervention than for late treatment. The reason for this difference might be that patients starting early biologic therapy for UC are more likely to have severe disease. Indeed, we observed substantial differences in serologic inflammatory disease severity between early and late initiators of biologic therapy.

In addition to efficacy, the safety profile should also be considered. In our study, early intervention with biologic and conventional step-up management had similar proportions of adverse events and colectomy. However, the result should be interpreted with caution because the long-term safety beyond 52 weeks was unknown, and some patients in our study did not reach the end of follow-up.

Despite our findings, it is essential to recognize several limitations of our study. First, although there are no statistic differences in Mayo score, inflammatory index and nutritive index at week 14 and 52, a lack of fecal calprotectin data, which is a useful surrogate marker of gastrointestinal inflammation, restricted our analysis. Second, the follow-up period of some patients was less than 52 weeks, which may have caused a certain bias in the efficacy and safety evaluation over the 52-week period. Last, given the retrospective nature of our data collection, there is an inherent need for prospective trials to more robustly determine the efficacy and safety of early intervention.

In conclusion, our multicenter, retrospective cohort study found that patients starting early biologic therapy within 3 years of UC diagnosis had similar rates of steroid-free clinical remission, clinical remission, mucosal healing, and the risk of adverse events compared to late initiators in moderate UC patients.

## Supplementary Material

Suppl 1 Clinical efficacy of early and late biologic intervention at week 14 and week 52.

## Data Availability

The data supporting the findings of this study are available from the corresponding author upon reasonable request.
