# Supplementary material for: Clinical Efficacy of Early and Late Vedolizumab or Infliximab Interventions in Moderate Ulcerative Colitis: A Multicenter, Retrospective Cohort Study
Source: Gastroenterology Res. 2026 Jan 4;19(1):35–42. doi: 10.14740/gr2092 (PMC12978406; doi:10.14740/gr2092)
Supplement: Suppl 1 — Clinical efficacy of early and late biologic intervention at week 14 and week 52. [file gr-19-01-035-s001.docx]

**Suppl 1.** Clinical efficacy of early and late biologic intervention at week 14 and 52

| **Time** |  | **Late** | **Early** | **P value** | **OR value** | **95% CI** |
| --- | --- | --- | --- | --- | --- | --- |
| 14 weeks | Steroid-free clinical remission | 70.2% (59/84) | 80.0% (32/40) | 0.250 | 1.70 | 0.69-4.19 |
|  | Clinical remission | 61.9% (52/84) | 67.5% (27/40) | 0.545 | 1.28 | 0.58-2.83 |
|  | Mucosal healing | 15.5% (13/84) | 17.5% (7/40) | 0.775 | 1.16 | 0.42-3.17 |
| 52 weeks | Steroid-free clinical remission | 77.1% (37/48) | 93.8% (15/16) | 0.267 | 4.46 | 0.53-37.65 |
|  | Clinical remission | 72.9% (35/48) | 93.8% (15/16) | 0.163 | 5.57 | 0.67-46.51 |
|  | Mucosal healing | 47.9% (23/48) | 56.3% (9/16) | 0.564 | 1.40 | 0.45-4.36 |
